# Current and potential distribution of the ectomycorrhizal fungus *Suillus lakei* ((Murrill) A.H. Sm. & Thiers) in its invasion range

**DOI:** 10.1007/s00572-018-0836-x

**Published:** 2018-05-15

**Authors:** Marcin Pietras, Monika Litkowiec, Joanna Gołębiewska

**Affiliations:** 1 Institute of Dendrology Polish Academy of Science, Parkowa 5, 62-035 Kórnik, Poland; 2 Department of Plant Taxonomy and Nature Conservation, University of Gdańsk, Wita Stwosza 59, 80-308 Gdańsk, Poland

**Keywords:** *Suillus lakei*, Ectomycorrhiza, Foreign fungus, Fungal biogeography, Ecological niche modeling

## Abstract

*Suillus lakei* is an ectomycorrhizal fungus native to North America and known in Europe, South America, and New Zealand. This contribution aims to illustrate the worldwide biogeography of *S. lakei* based on sporocarp records. Species distribution modeling was used to assess the suitable niche distribution of *S. lakei*, based on the climatic variables as well as distribution of its ectomycorrhizal partner, Douglas fir. In general, distribution of suitable niches of *S. lakei* greatly overlaps with the distribution of Douglas fir in North America. By spatial distribution modeling, we found that the precipitation of the coldest quarters, isothermality, and annual mean temperature are important factors influencing the potential distribution of *S. lakei*. Nevertheless, the most crucial factor limiting expansion of *S. lakei* in its invasion range is Douglas fir occurrence. This factor reached an 86.4% contribution for the *S. lakei* species distribution model. Additionally, we compare the aboveground and belowground presence of *S. lakei* based on surveys in the field. Our study shows that even extremely low abundance of ectomycorrhizas can open the possibility of using an ectomycorrhiza survey for their quantification as a good indicator of the presence of *S. lakei* in field conditions. Both sporocarps and ectomycorrhizas occurred only in gardens, where Douglas fir seedlings were outplanted at the beginning of the 1990s as an ornamental plant. Presumably, international trade of ornamental plants was one possible route of introduction of *S. lakei* to Poland.

**Electronic supplementary material:**

The online version of this article (10.1007/s00572-018-0836-x) contains supplementary material, which is available to authorized users.

## Introduction

Biological invasions have caused great losses of species diversity around the world. Therefore, from an ecological point of view, the introduction and spread of foreign and invasive organisms is one of the most important problems in nature conservation (Vitousek 1996). For example, the rapid expansion of the introduction of organisms in America began about 500 years ago, soon after its colonization by Europeans. Since then, about 50,000 alien species have been introduced to North America (Pimentel et al. 2000). Studies on anthropogenic introductions are focused on animals and vascular plants (Desprez-Loustau et al. 2007), which have been introduced to forestry and urban habitats.

One of the most planted tree species cultivated outside its natural range is Douglas fir (*Pseudotsuga menziesii*). It produces high-value timber and thus is considered the most economically important tree species in the world (Eckenwalder 2009). It is native to western regions of North America, where it dominates in wide-ranging forests, from the Rocky Mountains to the Pacific coast, often forming single-species forests typical for these regions (Farjon 1990). Because of its fast growth, rapid wound closure, and good wood properties, Douglas fir has also been cultivated outside its native range, in Europe and South America, for almost 200 years (Knoerzer and Reif 2001; Essl 2005). In Europe, Douglas fir is a commonly planted tree cultivated in France (427,000 ha), Germany (241,000 ha), in the British Isles (45,000 ha), and smaller areas in Denmark (5690 ha), Netherlands (16,000 ha), and Switzerland (2540 ha). In Poland, Douglas fir acreage has reached about 4852 ha (Chałupka 2014), but Douglas fir has never been cultivated for forestry in Poland. Since the beginning of the twentieth century, Douglas fir was also introduced to New Zealand. Nowadays, its plantations cover more than 100,000 ha and constitute the second most planted alien tree species in the country, after radiata pine. Douglas fir is obligatorily associated with ectomycorrhizal (ECM) fungi, requiring mycorrhizal association for its growth and development. This tree species forms ECM associations with as many as 2000 species of fungi (Trappe 1977), of which more than 25% are estimated to associate only with Douglas fir (Molina et al. 1992), including *Pseudotsuga*-specific lineages of suilloid fungi (Wood et al. 2015).

Three major strategies of invasion have been proposed (Dickie et al. 2010). First, *novel associations* are defined as mutualisms between trees and ECM fungi from different parts of the planet, which did not occur earlier because of geographical isolation. Second, *cosmopolitan associations* may occur between invasive trees and some common fungal taxa regarded as cosmopolitan (e.g., Moora et al. 2011). A third possibility is *co-invasion*, where a plant is introduced into a novel range with mutualists from its natural range. Although a large body of literature has been published regarding ECM fungi associated with Douglas fir, relatively little is known about the fungi that co-invaded with Douglas fir outside its natural range. This knowledge gap is particularly worrisome in relation to some Douglas-fir-specific fungal species, such as suilloid fungi, introduced outside their natural range: to New Zealand, South America, and Europe (Vellinga et al. 2009 and references therein, Wood et al. 2015).

*Suillus lakei* is one fungus typically associated with Douglas fir. This fungus is a commonly recorded ECM partner of Douglas fir in North America. The presence of *S. lakei* has been also documented outside its natural range: in South America and Europe and in Australia and New Zealand (Vellinga et al. 2009 and references therein), as a result of co-invasion of *S. lakei* with Douglas fir. Recently, *S. lakei* fruit bodies have been recorded also in central Poland (Usewicz 2012; Tylkowski 2013; Szczepkowski and Olenderek 2017).

The most important problems in studies concerning the invasion of fungi are reconstruction of a possible route of its introduction into new areas on the one hand and, on the other hand, assessment of the risk and possibility of further expansion of the fungal species in its invasion range.

There are numerous papers providing species distribution models for animal and plant species, but this approach is very rarely used for fungi. So far, modeling tools have been used in studies explaining the expansion of fungus-like oomycote plant pathogens: *Phytophtora ramorum* and *P. kernoviae* in Great Britain (Purse et al. 2013). In the case of fungi sensu stricto, modeling tools have been used to show the expansion of only one multiple-host ECM fungus, *Amanita phalloides* (Wolfe et al. 2009). Nevertheless, the knowledge of interactions between mycorrhizal fungi and Douglas fir in Europe is rudimentary, so further investigations are needed.

In this study, we have used species distribution modeling, a field survey, and molecular analysis, to evaluate its potential invasion range and to assess the current distribution and frequency of *Suillus lakei* in Poland. Thus, the following questions have been addressed.

- What is the current distribution of *S. lakei* and—based on climatic variables and Douglas fir occurrence—what is the potential range of this species?
- Which climatic factors are the most important in the expansion of *S. lakei*?
- How frequent and abundant is *S. lakei* in Poland, based on a survey of sporocarps and mycorrhizas?

## Materials and methods

### Database preparation and current distribution

The dataset of 93 records (49 for North America and 44 for invasion range, Appendix 1) was created. The occurrence data for native range of *Suillus lakei* were collected from Mycology Collections Portal (mycoportal.org) searching among preserved specimens. The assessment of distribution in invasion range was based on the literature, specimens preserved in herbaria, and our own data collected during field studies.

In the created model, we take into account the data concerning the distribution of Douglas fir as an ECM partner of *S. lakei*. Thus, we also downloaded occurrence data of Douglas fir accessible in GBIF (GBIF.org (2 January 2018)), searching among preserved specimens only. We assumed that the population from 55° N (central British Columbia) to 19° N (north of Mexico) and from the Pacific coast to the Rocky Mountains represent the natural range of Douglas fir in North America (Hermann and Lavender 1990). We also gathered data outside the natural range, from South America, Europe, Australia, and New Zealand. The georeferenced *S. lakei*, as well as Douglas fir, were considered independent when they were located at least 5000 m apart and, in case of Douglas fir, were observed after 1950.

### Potential distribution of *Suillus lakei* in Europe

**Fig. 1:**
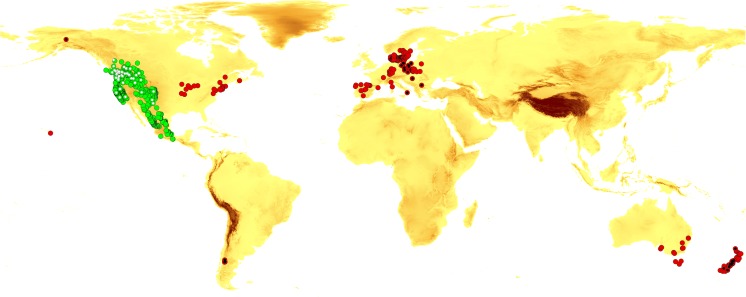
Native (white circles) and invasive (black circles) localities of *Suillus lakei*, on the background of Douglas fir records in native (green circles) and invasive range (red circles)

Models of suitable niche distributions were created separately for *S. lakei* and Douglas fir using MaxEnt 3.3.2 software (Philips et al. Phillips et al. 2006). This method gives an opportunity to determine the climatic variables linked with records in the natural range and to project its range to Europe based on areas where those variables are most similar. Input data were 12 climatic variables in 2.5 arc minutes (± 21.62 km^2^ at the equator, Table 1, Hijmans et al. 2005). Seven climatic variables were removed from analysis because of their significant correlation (above 0.9) as evaluated by the Pearson’s correlation coefficient calculation computed using ENMTools v1.3 (Kolanowska 2013; Kukwa and Kolanowska 2016). Additionally, Douglas fir occurrence data were used to assess distribution of *S. lakei* in the present time. In total, 93 different locations (49 for native range and 44 for invasion range, Appendix 1) of *S. lakei* and 1147 records of Douglas fir (729 for native range and 418 outside native range, Appendix 2) were included in the MaxEnt analysis (Fig. 1). Predicted distribution of suitable niches of Douglas fir was assessed using climatic variables and altitudinal data (Alt). To assess the potential distribution of *S. lakei*, two models were created. In the first, only climatic variables were used, and in the second, Douglas fir occurrence data were added to analysis. The maximum iteration number was set to 10,000 and the convergence threshold to 0.00001. For each run, 20% of the data were used and set aside as test points. The “random seed” option was used, which provided a random test partition and background subset for each run. Each run was performed as a bootstrap with 1000 replicates, and the output was set to logistic. All operations on GIS data were carried out on ArcGis 9.3 (ESRI). The model was evaluated using the most common metrics, i.e., area under the curve (AUC), where 1 indicates a perfect model, and values of more than 0.9 indicate a high performance of the model.

**Table 1:** Bioclimatic variables used in the ENM analysis

Code	Variable
alt	Altitude*
bio1	Annual mean temperature
bio2	Mean diurnal range = mean of monthly (max temp − min temp)
bio3	Isothermality (bio2/bio7) (*100)
bio4	Temperature seasonality (standard deviation *100)
bio5	Max temperature of warmest month
bio8	Mean temperature of wettest quarter
bio12	Annual precipitation
bio13	Precipitation of wettest month
bio14	Precipitation of driest month
bio15	Precipitation seasonality (coefficient of variation)
bio18	Precipitation of warmest quarter
bio19	Precipitation of coldest quarter

*Used only to create Douglas fir species distribution model

### Field survey

In Poland, for the first time in 2012, *S. lakei* was reported in a private garden, growing near ~25-year-old Douglas firs (Pruszcz). After that observation, five seed orchards of Douglas fir of a similar age were chosen to estimate the possible occurrence of *S. lakei*. Altogether, 13 sites were investigated (Table 2), representing gardens, plantations (including seed orchards), and mature seed stands. In each study site, sporocarps (if present) and 10 soil samples with Douglas fir roots were taken for molecular analysis once or several times between 2012 and 2015.

**Table 2:** Localities and characteristic of surveyed study sites

Name	Longitude	Latitude	Type	Age	Source
Nowe Ramuki	20.51667	53.65	Seed stand	176	Chylarecki 2004
Zdroje	16.3908	50.3726	Seed stand	165	Chylarecki 2004
Czaplinek	16.399	53.6748	Seed stand	164	Chylarecki 2004
Lutówko	17.5	53.616667	Seed orchard	15	http://www.bnl.gov.pl
Gniewkowo	18.366667	52.883333	Seed orchard	25	http://www.bnl.gov.pl
Łopuchówko	17.033333	52.55	Seed orchard	17	http://www.bnl.gov.pl
Piaski	17.0028	51.9963	Plantation	20	Personal information
Kórnik	17.0634	52.24	Plantation	30	Personal information
Poznan I	16.8978310,	52.4373857	Garden	22–25	Personal information
Poznan II	16.8990970,	52.4472347	Garden	25	Personal information
Pruszcz	18.2123280	53.3399958	Garden	20–25	Usewicz 2012
Splawie	17.0136166,	52.3542418	Garden	20	Tylkowski 2013

Tree roots from each soil sample (10 per site) were washed with tap water to remove particles. Morphological typing of the ECM root tips was performed under a stereomicroscope at 10–60× magnification. ECMs were separated into morphotypes based on macroscopic features. Suilloid morphotype was distinguished based on the following criteria: (1) white, thin mantle with a wooly, dense, pinkish mycelium on the surface; (2) dichotomous to irregular ramification of ectomycorrhizas; (3) a white to pinkish, usually abundant, extramatrical mycelium; (4) light brown, filamentous rhizomorphs. Only suilloid mycorrhizas, which might represent *S. lakei*, were counted, placed in Eppendorf tubes, and preserved in a refrigerator (at − 4 °C) for molecular identification.

### Molecular methods

Both sporocarps and mycorrhizas of *S. lakei* were subjected to molecular analyses. Total DNA was extracted from a single ECM root tip or from the small part of a sporocarp (ca 5 × 2 × 2 mm taken from the cap) using a Plant and Fungi DNA Purification Kit (Eurx) following the standard protocol. The PCR cocktail consisted of 4 ml of DNA extract, 0.5 ml of each primer (10 nM ITS5 and ITS4 (White et al. 1990)), and 5 ml of Type-it Microsatellite PCR Kit (Qiagen). PCR was carried out in the following thermocycling conditions: the initial 15 min at 95 °C, followed by 35 cycles at 95 °C for 30 s, 55 °C for 30 s, and 72 °C for 1 min, and a final cycle of 10 min at 72 °C. PCR products were estimated by running a 5-ml DNA amplicon on 1.5% agarose gel for 30 min. The PCR products were sequenced using ITS4 primers at the Laboratory of Molecular Biology of Adam Mickiewicz University (Poznań). The obtained sequences were verified visually on chromatograms using BIOEDIT. Nuclear ITS sequences obtained in this study have been deposited in GenBank (with accession numbers KY883336-KY883343; even numbers for sporocarps, uneven for ectomycorrhizas).

## Results

### Potential distribution of Douglas fir and *Suillus lakei*

**Fig. 2:**
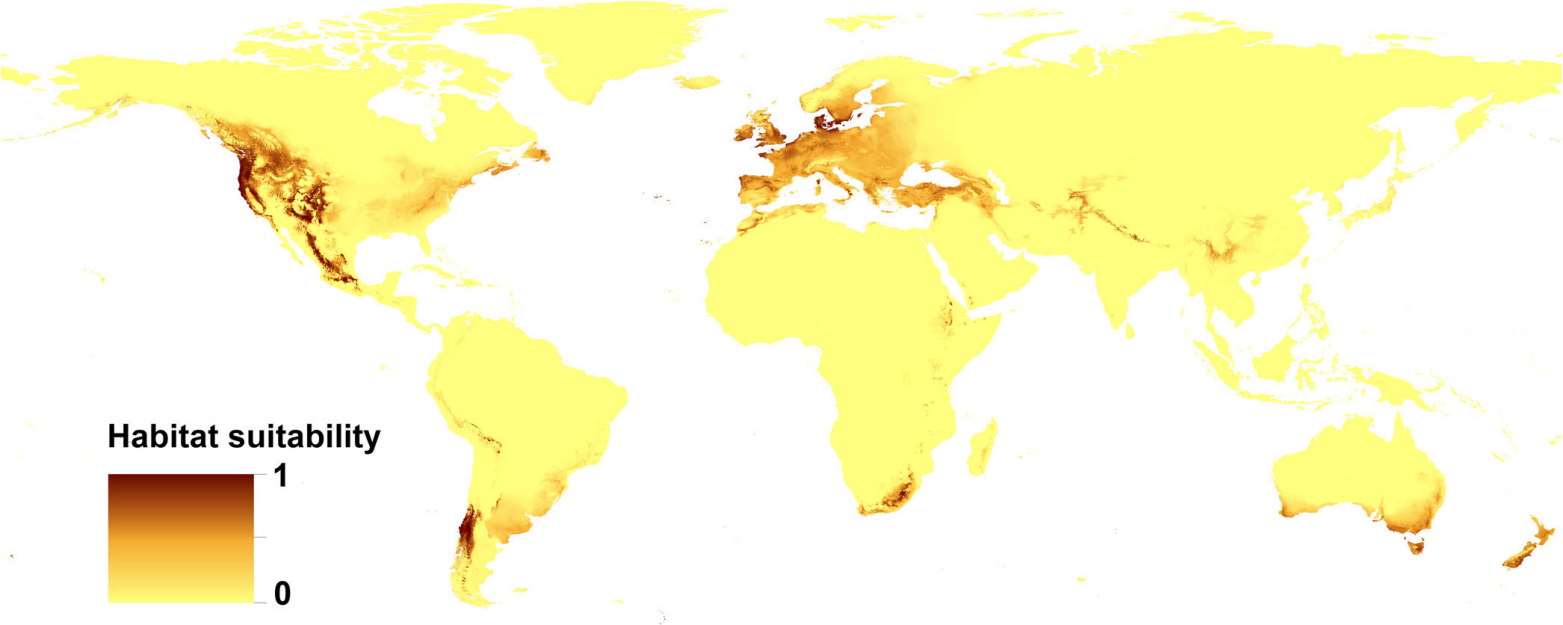
Present distribution of the suitable habitats of Douglas fir

**Fig. 3:**
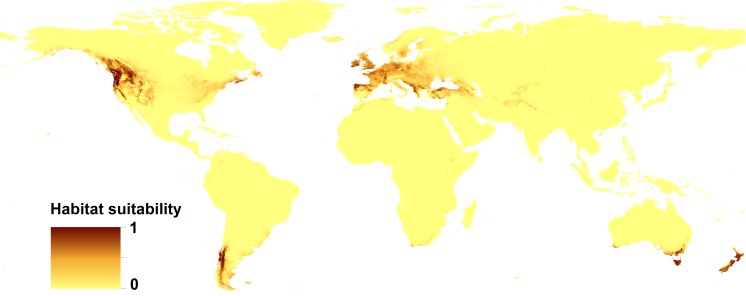
Present distribution of the suitable habitats of *Suillus lakei* (based on climatic variables only)

**Fig. 4:**
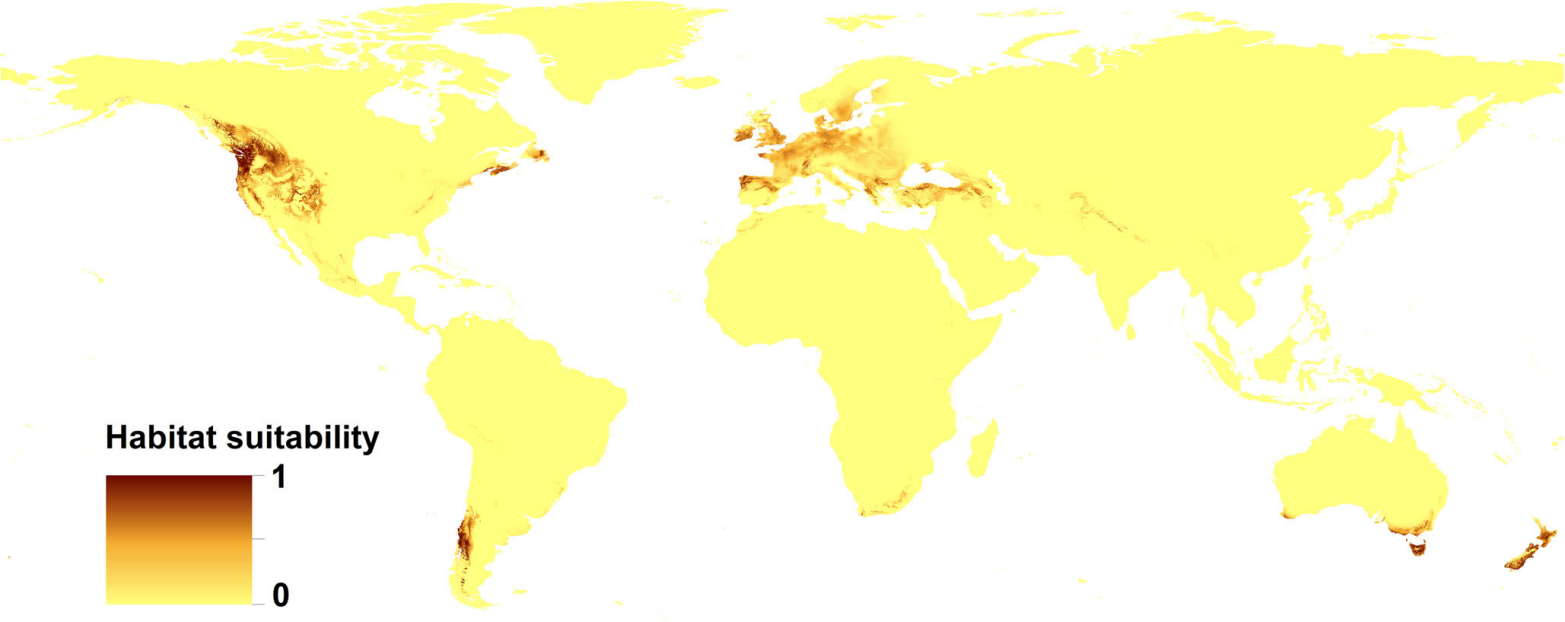
Present distribution of the suitable habitats of *Suillus lakei* (based on climatic variables and Douglas fir occurrence data)

All the models received high AUC scores, which indicates the high performance of the models (Table 3). The distribution of suitable niches of Douglas fir greatly overlaps with its distribution in native range (areas between British Columbia in Canada and central Mexico on the western part of North America (Fig. 1 and Fig. 2)). In invasive range species, distribution model of Douglas fir shows the wide range of suitable habitats in western, central, and southern Europe (to the Anatolian Peninsula in the east), including the British Islands as well as the southern edge of the Scandinavian Peninsula. In the Southern Hemisphere, the suitable habitats include parts of southern South America (the mountains in the north part of Chile and Argentina), the southern coasts of Australia, all of Tasmania, and both isles of New Zealand, as well as southern edge of Africa. The model created for *S. lakei* based on climatic variables only is compatible with the potential range of Douglas fir, with smaller areas occupied in North America (excluding Mexico). In the invasive range, the prediction for *S. lakei* is even stronger than for its host plant (Fig. 2 and Fig. 3). A combined model created based on both Douglas fir occurrence data and climatic variables (Fig. 4) shows weaker prediction for *S. lakei* occurrence in the native and invasive range. For Europe, this model also predicts some reduction of the suitable habitats, especially in marginal areas of the invasive range.

**Table 3:** Estimates of relative contributions of the environmental variables to the Maxent model created for Douglas fir and *S. lakei*

Model		
Douglas fir (altitudinal and climatic data)	*Suillus lakei* (climatic data)	*Suillus lakei* (climatic data and Douglas fir occurrence)
AUC = 0.948 (SD = 0.001)	AUC = 0.948 (SD = 0.003)	AUC = 0.951 (SD = 0.002)
Annual mean temperature (39.5)	Precipitation of coldest quarter (26.6)	Douglas fir occurrence (86.4)
Temperature seasonality (31.9)	Isothermality (26.2)	Precipitation of coldest quarter (4.3)
Precipitation of coldest quarter (7.2)	Annual mean temp. (25.1)	Precipitation of driest month (2.9)

The percent contribution is given in parenthesis

The generated models show different predictions (Table 3). The limiting factors differ between the generated models created for Douglas fir and *S. lakei*. The annual mean temperature, temperature seasonality, and precipitation of the coldest quarter are the most important factors limiting the potential distribution of Douglas fir. There was a particularly large difference in the importance of variables related to the two models created for *S. lakei*. For the model that only considers climatic variables, the most decisive factors were temperature of coldest quarter, isothermality, and annual mean temperature. In the model created based on both climatic variables and Douglas fir records, the most important limiting factor influencing the present potential distribution of *S. lakei* was Douglas fir occurrence (Table 3).

### Field survey of *Suillus lakei* sporocarps and ectomycorrhizas

After the first record of *S. lakei* in Poland in 2012 (Usewicz 2012), this fungus was also observed in the neighboring three localities, all of them about 150 km west of Poznań. Previously, *S. lakei* was reported in other European countries: Germany, Slovenia, the Czech Republic, and Italy (see Supplementary Material). Of the 12 surveyed sites in Poland, sporocarps of *S. lakei* were recorded only in four gardens, in contrast to five Douglas fir seed orchards, plantations, and mature seed stands, located in forests, where sporocarps of this species were absent. The highest sporocarp densities were reached on the Pruszcz site. Regarding the belowground survey, all mycorrhizas representing the suilloid type (distinguished during morphotyping) were identified as *S. lakei*. Similar to sporocarps, ectomycorrhizas of *S. lakei* were found only in gardens and made up a small percentage of the total number of ECM root tips. In the soil cores taken below respective sporocarps, root tips colonized by *S. lakei* were more abundant and accounted for a substantial proportion of the ECM community, as much as 20% of the relative abundance of ECM root tips. However, the frequency of ectomycorrhizas formed by *S. lakei* differed between sites. The highest ECM frequency was encountered on the Pruszcz site (100%) and the lowest on the Poznan II site (20%). The frequency of ECMs on the Splawie and Poznan I sites reached 50 and 70%, respectively. Other common ECM morphotypes detected during this research were the *Wilcoxina*/*Trichophaea* type, *Lactarius* type, and thelephoroid fungi (data not shown).

## Discussion

The introduction of *S. lakei* into Europe can be a valuable component of the discussion on the expansion of non-pathogenic fungi. The close association between Douglas fir and *S. lakei*, as a fungus with a high specificity for *Pseudotsuga* spp., limits the investigation to the areas where Douglas fir occurs. Distribution of suitable niches of Douglas fir greatly overlaps with the current range and potential distribution of this tree in North America (Boiffin et al. 2017) and Europe (Da Ronch et al. 2016; Dyderski et al. 2017). The extended range of Douglas fir in Europe can be related to the changing niche preference or alternatively to natural or human-driven selection (Boiffin et al. 2017). In our study, we showed for the first time the potential distribution of ECM fungus using climatic variables on the background of distribution of its ECM plant partner. Compared with Douglas fir, the *Suillus lakei* potential range shows overlap in North America. In the invasion range, especially in Europe, the area of potential distribution of *S. lakei* is larger than that of Douglas fir acreage. In some regions where *S. lakei* has never been recorded (New Zealand North Island, the south coast of Australia and Tasmania), a strong prediction for its occurrence was detected. In these regions, different strategies of environmental management can be used, even when they are not very applicable on a large scale (e.g., blocking human vectors) or are harmful to the environment (e.g., Douglas fir forest removal) (Dickie et al. 2016). Our model also predicted that climatic conditions on the south coast of Africa are favorable for *S. lakei* occurrence, but the lack of Douglas fir in this region is a limiting factor for fungus spread. Therefore, the discontinuation of Douglas fir planting in new regions can stop the expansion of *S. lakei* in new areas. The differences between the occupied niches are reflected in the limiting factors for Douglas fir and *S. lakei*. Douglas fir grows under a wide variety of climatic conditions. The coastal region of the Pacific Northwest is characterized by a maritime climate with wet winters and cool, relatively dry summers. The central Rocky Mountains, where the climate is continental, have long and harsh winters and dry and hot summers. Our analysis shows that annual mean temperature and temperature seasonality are the most important limiting factors influencing the potential distribution of Douglas fir. In a recent paper published by Boiffin et al. (2017), annual temperature range and precipitation seasonality defined the climatic gradient that best sorted occurrences of Douglas fir in the native range from those of the European occurrences of Douglas fir. This study considered only European records of Douglas fir outside the native range, thus the lack of data from South America and New Zealand can be the reason for differences from our results. Similarly, Dyderski et al. (2017) showed that for European populations the most decisive factors for Douglas fir occurrence are the temperature’s annual range and the mean temperature of the warmest quarter. Our findings cannot be compared with results presented by Dyderski et al. (2017) because of methodological differences. Dyderski et al. (2017) created models to predict future changes in distribution of Douglas fir based on 19 climatic variables, in comparison with 13 variables (12 climatic and one altitude) in our study. Our analysis showed as well that the model of Douglas fir distribution did not reveal altitude as crucial factor for Douglas fir occurrence (0.7% of the contribution, data not shown). This is expected because the tree naturally occurs between an altitude of 0 and 3200 m above sea level (a.s.l.). Precipitation of the coldest quarter, isothermality and annual mean temperature were the most significant factors for *S. lakei* in the model created based on climatic variables only. For the model where Douglas fir occurrence data were added, this factor was crucial for *S. lakei* occurrence, reaching an 86.4% contribution (Table 3). Thus, the most important limiting factor for further expansion of *S. lakei* in the invasion range is the occurrence of its ectomycorrhizal partner, Douglas fir. The obtained results highlighted the climatic condition in expansion of alien fungi. In general, it is not surprising that the occurrence of fungi is strongly related to high precipitation because, generally, fungi prefer humid conditions (Krebs et al. 2008). Several authors have argued that the occurrence of epigeous fungi is linked to annual climatic conditions, such as the average annual or monthly precipitation (Eveling et al. 1990). Salerni et al. (2014) showed that production of high value truffles is positively correlated with the rainfall of the previous 3 months and, in general, with those of the autumn months prior to collection. For epigeous fungi, Taye et al. (2016) showed a strong influence of weather conditions on the appearance of the *Lactarius* group sporocarps and mushroom productivity in central Spain. Especially, changes in seasonal precipitation represented the main weather-related driver affecting sporocarp emergence and production. Precipitation and mean temperature of the driest month were major drivers of the occurrence of a saprobic fungus, *Clathrus archeri*, in its invasive range (Pietras et al. 2016). Our study confirms the assumption that climatic conditions should be regarded as a crucial factor in the invasion ecology of fungi.

Of all the plantations, seed orchards and mature seed stands of Douglas fir forests in Poland, eight were surveyed to assess the potential occurrence of *S. lakei*. The survey of sporocarps and mycorrhizas did not reveal the presence of *S. lakei* in the surveyed seed orchards and mature seed stands. All of the Polish records represent gardens located near forests and established approximately 25 years ago, where both sporocarps and ectomycorrhizas of *S. lakei* were found. Soil cores taken below sporocarps were characterized by a higher abundance of ECM root tips colonized by *S. lakei* (up to 20%), especially on the Pruszcz study site, where the highest number of sporocarps were found (data not shown). ECM fungi exist as a complex of ectomycorrhizae and sporocarps that are connected with and develop from extramatrical mycelia. Previous work demonstrated that the abundance of belowground mycelia of other ECM fungi (e.g., the closely related *S. grevillei*) are not connected with the number and distribution of sporocarps (Zhou et al. 2001). Koide et al. (2005) have suggested that ECM fungal species differ in their spatial distribution on root tips, and that root tip and mycelia views of the community are different. In the case of another North American fungus, *Suillus pungens*, Gardes and Bruns (1996) show that it commonly produces fruiting bodies but is a rare component belowground. Similarly, mycelium of two different *Suillus* species were never encountered in soil on plantations of *Pinus patula*, where sporocarps of those species were found in abundance (Natarajan et al. 1992). In our study, no statistically significant association between ECM root tips of *S. lakei* and sporocarp formation could be found because we did not focus on mushroom site productivity, seasonality, and distribution of sporocarps within the study sites. Even though our results confirm that, within the *Suillus* group, ECMs were rare and scattered belowground, their sporocarps were frequent and abundant aboveground in gardens. However, our study shows that the detectable presence of ectomycorrhizas (even at an extremely low abundance) could open the possibility of using methods for their quantification as a good indicator of the presence of *S. lakei* in field conditions, especially in the case of single records, where a less time-consuming analysis is required. The survey of both ectomycorrhizas and sporocarps did not reveal the presence of *S. lakei* in seed orchards located in forests. An explanation for this is that the analyzed seed orchards were established from Douglas fir seedlings (cultivated from seeds taken from old Polish seed stands) and were grown in bare-root forest nurseries in Poland, in contrast to gardens, where the presence of *S. lakei* has been revealed. Since the beginning of the 1990s, Douglas fir was commonly used as an ornamental plant. At that time, most of the planting stock was imported as seedlings in pots from Western Europe, mainly from Germany. According to a review paper by Vellinga et al. (2009), several ECM fungi associated with Douglas fir were detected in Central and Western Europe, including *S. lakei*. As mentioned above, in Poland, *S. lakei* was recorded for the first time in 2012 (Usewicz 2012) in a private garden, where the outplanted Douglas fir seedlings were imported in pots from Germany (Usewicz, private communication). In a recent study presenting up-to-date data on the distribution of *S. lakei* in central Poland, sporocarps were discovered on private properties overgrown with young Douglas firs (Szczepkowski and Olenderek 2017). Information about the origin of the Douglas fir growing on private gardens is not available for most localities where *S. lakei* was found; however, this observation suggests that the international trade of ornamental plants can be one possible route of introduction of ECM fungi.

## Electronic supplementary material

Appendix 1 *Suillus lakei* occurrence data (CSV 2 kb)

Appendix 2 Douglas fir occurrence data (CSV 36 kb)

Supplementary material : List of localities of *Suillus lakei* used in the modeling (XLSX 18 kb)
